# Developmental plasticity of Arabidopsis hypocotyl is dependent on exocyst complex function

**DOI:** 10.1093/jxb/erz005

**Published:** 2019-01-14

**Authors:** Edita Janková Drdová, Martina Klejchová, Karel Janko, Michal Hála, Hana Soukupová, Fatima Cvrčková, Viktor Žárský

**Affiliations:** 1 Institute of Experimental Botany, Academy of Sciences of the Czech Republic, Prague 6, Czech Republic; 2 Department of Experimental Plant Biology, Faculty of Science, Charles University, Prague 2, Czech Republic; 3 Institute of Animal Physiology and Genetics, Academy of Sciences of the Czech Republic, Liběchov, Czech Republic

**Keywords:** *Arabidopsis thaliana*, auxin, collet, etiolated hypocotyl, exocyst, root–hypocotyl junction, starch accumulation

## Abstract

The collet (root–hypocotyl junction) region is an important plant transition zone between soil and atmospheric environments. Despite its crucial importance for plant development, little is known about how this transition zone is specified. Here we document the involvement of the exocyst complex in this process. The exocyst, an octameric tethering complex, participates in secretion and membrane recycling and is central to numerous cellular and developmental processes, such as growth of root hairs, cell expansion, recycling of PIN auxin efflux carriers and many others. We show that dark-grown Arabidopsis mutants deficient in exocyst subunits can form a hair-bearing ectopic collet-like structure above the true collet, morphologically resembling the true collet but also retaining some characteristics of the hypocotyl. The penetrance of this phenotypic defect is significantly influenced by cultivation temperature and carbon source, and is related to a defect in auxin regulation. These observations provide new insights into the regulation of collet region formation and developmental plasticity of the hypocotyl.

## Teaser

Etiolated hypocotyls of exocyst mutants exhibit a defect in hypocotyl plasticity manifested as ectopic collet-like structure formation, associated with deviations in auxin signaling and starch accumulation.

## Introduction

Germinating seedlings of many plant species require mechanical support and stabilization provided by their roots for proper development. Before the primary root fully develops, stabilization is provided by the collet hairs growing from the collet (root–hypocotyl junction) region. The collet region is an important transition zone between soil and atmospheric environments and represents an interface between two structures that strikingly differ in their external and internal anatomy, i.e. the root, which has one cortical cell layer, and the hypocotyl with two cortical layers (Lin and Schiefelbein, 2001). Root and hypocotyl also differ in plastid status, with prevailing chloroplasts in the hypocotyl and amyloplasts in roots, as well as in the presence of cuticle on the hypocotyl, but not on the root.

In spite of the collet region’s crucial role in the plant development, only a few studies have investigated its establishment. Analyses of Arabidopsis embryogenesis (Scheres *et al.*, 1994, 1996; Lin and Schiefelbein, 2001) showed that the collet region is established early in the heart and post-heart stage embryos concomitantly with the root and hypocotyl. The expression of the transcription factor GLABRA2 (GL2), which suppresses root hair formation, is also lower in the collet region already in the torpedo stage of embryogenesis and is missing completely in the later stages. This indicates that the identity of hair producing cells is determined already at an early stage of embryogenesis (Lin and Schiefelbein, 2001). Collet hair development was recently studied only with respect to endoreduplication status (Sliwinska *et al*., 2012, 2015).

Here we report that development of dark-grown hypocotyl can be modified and formation of additional ectopic collet-like structures can by induced by modulation of auxin transport through knock-out mutations in the exocyst complex.

The exocyst, an octameric protein complex consisting of Sec3, Sec5, Sec6, Sec8, Sec10, Sec15, Exo70, and Exo84 subunits (see Wu and Guo, 2015), participates together with other regulatory proteins in tethering and docking of secretory vesicles to discrete domains of the plasma membrane. The exocyst is ancient and ubiquitous among eukaryotes and the presence of all eight subunits has been documented also in plants (Synek *et al.*, 2006; Koumandou *et al.*, 2007; Hála *et al.*, 2008). Comparative analyses of genomes uncovered an extreme multiplication of Exo70 paralogs in land plants (Synek *et al.*, 2006, Cvrčková *et al.*, 2012; Rawat *et al.*, 2017).

Arabidopsis exocyst subunit loss of function mutants display various morphological or cell polarity defects, such as overall compromised growth, defective cell plate formation during cytokinesis, impaired elongation of pollen tubes and root hairs, and altered seed coat deposition and trichome maturation (Cole *et al.*, 2005; Wen *et al.*, 2005; Synek *et al.*, 2006; Hála *et al.*, 2008; Fendrych *et al.*, 2010; Kulich *et al*., 2010, 2015). Exocyst subunits EXO70A1 and SEC8 are involved in recycling of auxin transporters PIN1, PIN2, and the brassinosteroid receptor BRI1 to the plasma membrane, consistent with the plant exocyst being a general regulator of plasma membrane protein recycling (Drdová *et al.*, 2013). Mutant *exo70A1* plants exhibit stunted growth, perturbed apical dominance, and almost sterile inflorescences (Synek *et al.*, 2006; Drdová *et al.*, 2013). Etiolated *exo70A1* seedlings have shorter hypocotyls than wild type (WT) plants due to reduction in the number and length of cells (Synek *et al.*, 2006). Reduced cell and organ elongation was also reported for *exo84b* and *sec8* mutants (Synek *et al.*, 2006; Hála *et al.*, 2008; Fendrych *et al.*, 2013).

In the present study, we show that etiolated seedlings of several exocyst mutants (*exo70A1*, *sec15b*, *exo84b-1*, and *sec8m3/LAT52::SEC8*) can form ectopic collet hair-like structures that are located above the normal collet region. This defect, which, to our knowledge, has never been observed in any Arabidopsis WT or mutants, is accompanied by changes in auxin response, impaired PIN3–green fluorescent protein (GFP) localization and ectopic starch accumulation in the affected region. These observations suggest an important role of the exocyst in environmentally regulated hypocotyl developmental plasticity related to polar auxin transport and signaling.

## Materials and methods

### Plant material

Mutant *Arabidopsis thaliana* L. Heynh lines *exo70A1-1*, *exo70A1-2* (Synek *et al.*, 2006), *sec8m3/LAT52::SEC8* (Cole *et al.*, 2005), and *exo84b-1* (Fendrych *et al.*, 2010) have been described previously, as well as lines expressing DR5::GUS (Ulmasov *et al.*, 1997), PIN3::PIN3-GFP (Žádníková *et al.*, 2010) and GL2::GFP (Lin and Schiefelbein, 2001). T-DNA mutant line *sec15b-1*, SALK_130663 of the Columbia-0 ecotype obtained from the SALK Institute (Alonso *et al.*, 2003), and transposon insertional line *sec15b-2* of the Nossen-0 ecotype, RATM15-1183-1_H from Riken BRC (Ito *et al.*, 2002) were backcrossed to the Columbia-0 ecotype. The location of each insertion within the *SEC15b* gene (At4g02350) was verified by PCR with specific primers (see Supplementary Table S1; Supplementary Fig. S1A at *JXB* online).

### Culture conditions

Arabidopsis seeds were surface-sterilized for 10 min in 20% household bleach (Bochemie, Savo), rinsed three times with sterile distilled water, sown onto agar medium plates containing ½ Murashige and Skoog salts (Sigma-Aldrich) supplemented with 1% (w/v) sucrose (Fluka/Sigma-Aldrich) or mannitol (0.528%), vitamins, and 1.6% (w/v) plant agar (all Duchefa), buffered to pH 5.7, and stratified at 4 °C for 2 d in the dark. Seedlings were grown vertically in a climate chamber at 22 °C unless stated otherwise, either in the dark or under long day (16 h light–8 h dark) conditions.

### *SEC15b* mRNA detection using RT-PCR

To verify absence of the *SEC15* transcript in *sec15b* mutants by RT-PCR, 2-week-old seedlings from long day conditions were harvested and immediately frozen in liquid nitrogen. Total RNA was extracted using RNeasy Plant Mini Kit (Qiagen, Germantown, MD, USA) according to the manufacturer’s instructions. RNA (1 µg) was converted to cDNA by the Transcriptor High Fidelity cDNA Synthesis Kit (Roche, Mannheim, Germany) according to the manufacturer’s recommendations using an oligo-dT primer. The cDNA was amplified by PCR using a set of primers specific to the *SEC15B* gene; as an internal amplification and template control, PCR amplification of the constitutively expressed actin gene *ACT7* (At5g09810) with appropriate specific primers was used (Supplementary Table S1). An equal quantity of PCR product was loaded on 0.8% agarose gel (Supplementary Fig. S1B).

### Staining

For β-glucuronidase (GUS) staining, 5-day-old etiolated seedlings were vacuum-infiltrated with a solution consisting of 50 mM sodium phosphate buffer pH 7.2, 250 µM K_3_Fe(CN)_6_, 250 µM K_4_Fe(CN)_6_, 2% Triton X-100, and 1 mM 5-bromo-4-chloro-3-indolyl-β-D-glucuronic acid (X-GlcA, Duchefa) for 1 h and then incubated at 37 °C until color development.

For starch detection, 5-day-old etiolated seedlings were incubated for 5 min in Lugol’s solution (Sigma-Aldrich), followed by 10 min washing with water.

Improved propidium iodide staining was done as described in (Truernit *et al.*, 2008) with modifications (80 °C prewarmed ethanol was applied for 1 min and Hoyer’s solution was not used).

Cuticular defects were detected using an aqueous solution of 0.05% toluidine blue (Sigma-Aldrich) for 10 min, followed by washing with water.

### Microscopy and image analysis

Seedling morphology was documented with a binocular Leica S6D microscope with a digital camera attached. The chlorophyll autofluorescence and GUS, starch, or cuticular wax staining were documented using an Olympus BX-51 microscope with an Olympus DP50 camera attached. For observation of transversal sections, seedlings fixed in 5% agar were cut using a vibrating blade microtome to 150 µm sections and observed using an Olympus AX70 microscope.

Seedlings expressing GL2::GFP and propidium iodide-stained samples were observed using a Spinning Disc confocal microscope (Yokogawa CSU-X1 on the Nikon Ti-E platform, laser box Agilent MLC400, Zyla sCMOS camera by Andor). Full *z*-stack confocal images were 3D-projected. Image processing and stack projections were performed using Fiji software.

To quantify GUS staining intensity, 500 μm × 500 μm regions of interest (ROIs) were selected covering the ectopic collet-like region of each hypocotyl (or corresponding area in the case of WT) using Fiji. Each ROI was converted to 8 bit, binarized using the empirically determined threshold value of 0–80, and the area above threshold was determined. The same approach was used to quantitatively estimate starch accumulation except that the analysed ROI of the hypocotyl was 500 μm×1600 μm and the threshold was set to 0–40.

Seedlings expressing PIN3–GFP were observed using a Zeiss LSM 880 confocal scanning microscope equipped with a Zeiss C-Apochromat ×40/1.2 water-corrected lens.

### Statistics

To evaluate the effects of culture conditions on hypocotyl length, we used forward selection of generalized linear models with Gaussian error distribution. A series of *post hoc* pairwise Tukey tests with *P*-value correction for multiple testing was subsequently used to determine significance of treatment effects for each genotype.

To test for differences in the distribution of numbers of adventitious roots between mutant and WT plants in respective regions (collet versus hypocotyl), we used a generalized linear mixed effect model with Poisson error distribution implemented in the R library lme4; (Bates *et al.*, 2014), treating the number of adventitious roots as the dependent variable and genotype, plant region, and the interaction between both as explanatory variables. The fitted models incorporated a nested group effect of individual plants being nested within genotypes. The models were fitted separately to the dataset incorporating the *sec15b* mutant with its WT counterpart and to the dataset incorporating the *exo70A1* mutant with the respective WT counterpart. The significance of individual terms was tested with the lmerTest library of R.

## Results

### Etiolated hypocotyls of exocyst mutants produce an ectopic collet-like region and exhibit altered adventitious root induction

**Fig. 1.**
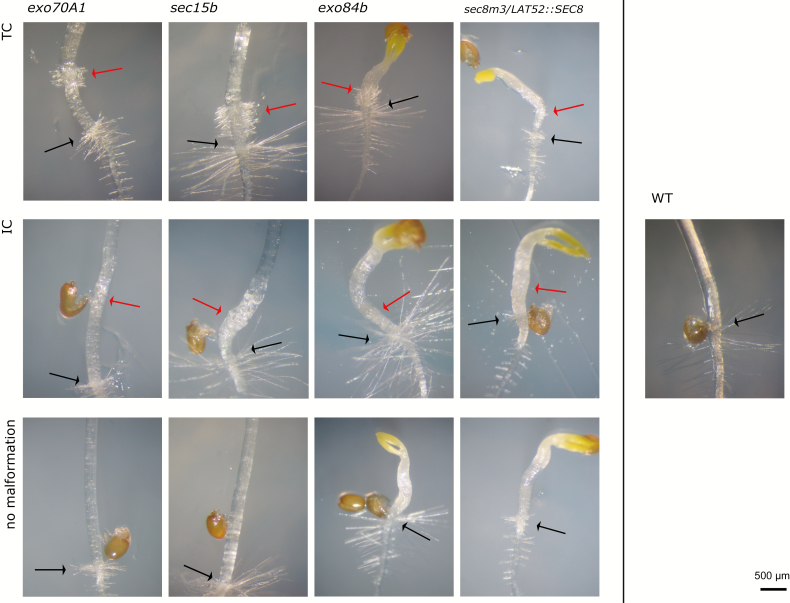
Phenotypic defects of etiolated hypocotyls in exocyst mutants. Typical phenotypic deviations—ectopic collet-like structures with or without developed hairs (phenotypes TC and IC) and shortened hypocotyls without malformations in 7-day-old *exo70A1*, *sec15b*, *exo84b*, and *sec8m3/LAT52::SEC8* mutant seedlings. Black arrows, collet hairs; red arrows, ectopic collet-like structures. A WT seedling is shown alongside for comparison.

All mutants defective in exocyst subunits analysed in this study (i.e. alleles *exo70A1-2*, *sec15b-1*, *exo84b-1*, and *sec8m3/LAT52::SEC8*) had notably shorter hypocotyls than WT plants when grown in the dark (i.e. etiolated) on sucrose-containing medium. However, apart from this defect, they often displayed additional developmental alterations easily distinguishable in 5- to 7-day-old plants (Fig. 1; Supplementary Fig. S2A). Namely, some seedlings formed a discrete region on their hypocotyl, which was located close above the collet and exhibited morphological features resembling the true collet, such as irregular cell expansion and hair formation on clustered trichoblasts. We will therefore refer to these aberrant structures as ‘ectopic collet-like regions’.

In general, we observed two major types of morphological malformations in *exo70A1* and *sec15b* mutants. The first category and the most severe phenotype we defined by formation of ectopic collet-like hairs in the proximity of root–hypocotyl junction, leading to an appearance of two collets (hereafter referred as ‘twin-collet’—TC). The second category, probably less severe, we defined by the existence of irregularly shaped epidermal cells without ectopic hairs (hereafter referred to as ‘irregular cells’—IC). We note that the aforementioned phenotypic malformations were never observed in WT plants or any other mutant.

In the *exo70A1-2* and *sec15b-1* mutants, the ectopic collet-like region was always separated from the regular collet by a region of apparently normal hypocotyl cells. The situation was more complicated in *exo84b-1* and *sec8m3*/*LAT52::SEC8* mutants where such a separation was not always apparent due to their extremely shortened hypocotyls, thereby complicating detection of the IC phenotype. Since the hairs formed at the ectopic collet-like region are much shorter than those at the normal collet in *exo84b-1*, we could clearly identify TC formation even in this extremely dwarfed mutant (Fig. 1; Supplementary Fig. S2A). On the other hand, regular collet hairs of *sec8m3*/*LAT52::SEC8* are as short as the ectopic hairs, making the TC phenotype less prominent. In further analyses, we therefore focused on the *exo70A1-2* (hereafter *exo70A1*) and *sec15b-1* (hereafter *sec15b*) mutants with well-pronounced phenotypic defects.

We noted that the penetrance of the above-described phenotypic defects varied among experiments, and subsequently found that the severity of the *sec15b* phenotypic deviation positively correlated with cultivation temperature. At 18 °C, most *sec15b* seedlings had short but otherwise morphologically normal hypocotyls but at 28 °C most plants had TC. Cultivation at 22 °C resulted in an intermediate proportion of phenotypic defects, with a considerable fraction of plants showing IC. In the *exo70A1* mutant, however, the hypocotyl defects were not significantly affected by the cultivation temperature (Fig. S2B).

To further investigate hypocotyl plasticity in exocyst mutants, we compared their ability to induce adventitious roots along the hypocotyls upon transition to light after 5 d of growth in the dark (Correa *et al.*, 2012; Verstraeten *et al.*, 2014). We found, as expected (Correa *et al.*, 2012), that WT plants formed adventitious roots along entire hypocotyl, especially in its upper part, with only a minority located in the collet. In contrast, *exo70A1* and *sec15b* mutants initiated most adventitious roots in the true collet region but rarely along the rest of the hypocotyl (for quantitative data see Supplementary Table S2).

### The ectopic collet-like region in *exo70A1* mutant exhibits altered internal anatomy

**Fig. 2.**
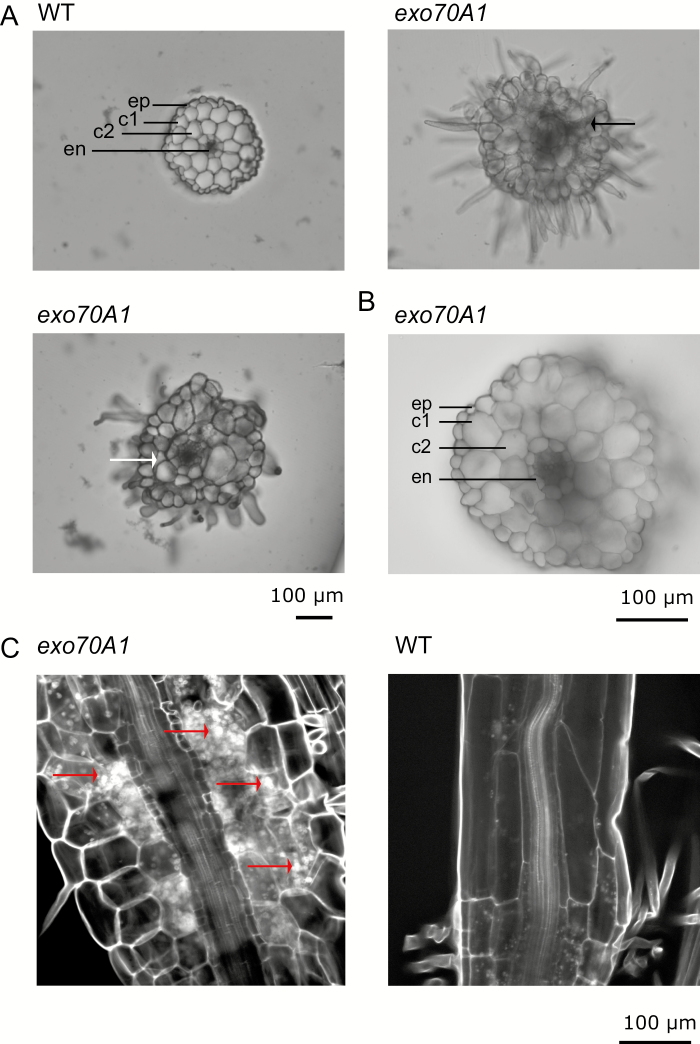
Anatomy of the ectopic collet-like region. (A) Transverse sections through the ectopic collet-like region of etiolated hypocotyl of 5-day-old *exo70A1* seedlings compared with WT hypocotyl anatomy. Note the adjacent position of ectopic-hair-carrying cells in the *exo70A1* mutants. Collapsed cells are indicated by a black arrow, incomplete two layers of cortical cells by a white arrow. (B) A section through the *exo70A1* mutant hypocotyl between the collet and the ectopic collet-like region, with nine cells in the endodermis and nine cells in the inner layer of cortex. c1, c2, layers of cortex; en, endodermis; ep, epidermis.

The ectopic collet-like structure in TC plants usually formed many hairs with hair-producing cells adjacent to each other, reminiscent of a normal WT collet rather than the root. Transversal sections through this structure of an *exo70A1* hypocotyl revealed irregular cell organization with enlarged cells in the epidermis, cortex, and endodermis. Collapsed cells and missing parts of one of the two cortex cell layers were sometimes observed. The number of cells in the endodermis and inner layer of cortex in some mutant plants varied compared with the WT, which always had two complete cell layers in the cortex with eight cells in both endodermis and the inner cortex layer (Fig. 2A). However, the region between the ectopic collet-like structure and the true collet displayed typical hypocotyl characteristics, i.e. two complete layers of cortex, although cells in this region were also enlarged and cell numbers in the cortex and endodermis were increased compared with the WT in some plants (two out of five sectioned hypocotyls; Fig. 2B). Optical longitudinal sections through the ectopic collet-like region of *exo70A1* revealed extremely shortened, irregularly shaped or collapsed cells in the endodermis and cortex, and expanded epidermal cells with ectopic hairs. Improved propidium iodide staining, applied to enhance cell wall contrast, further suggested accumulation of starch in the ectopic collet-like region of *exo70A1* mutants (Fig. 2C).

### Auxin and starch dynamics are altered in the ectopic collet-like region of *exo70A1* and *sec15b* mutants

**Fig. 3.**
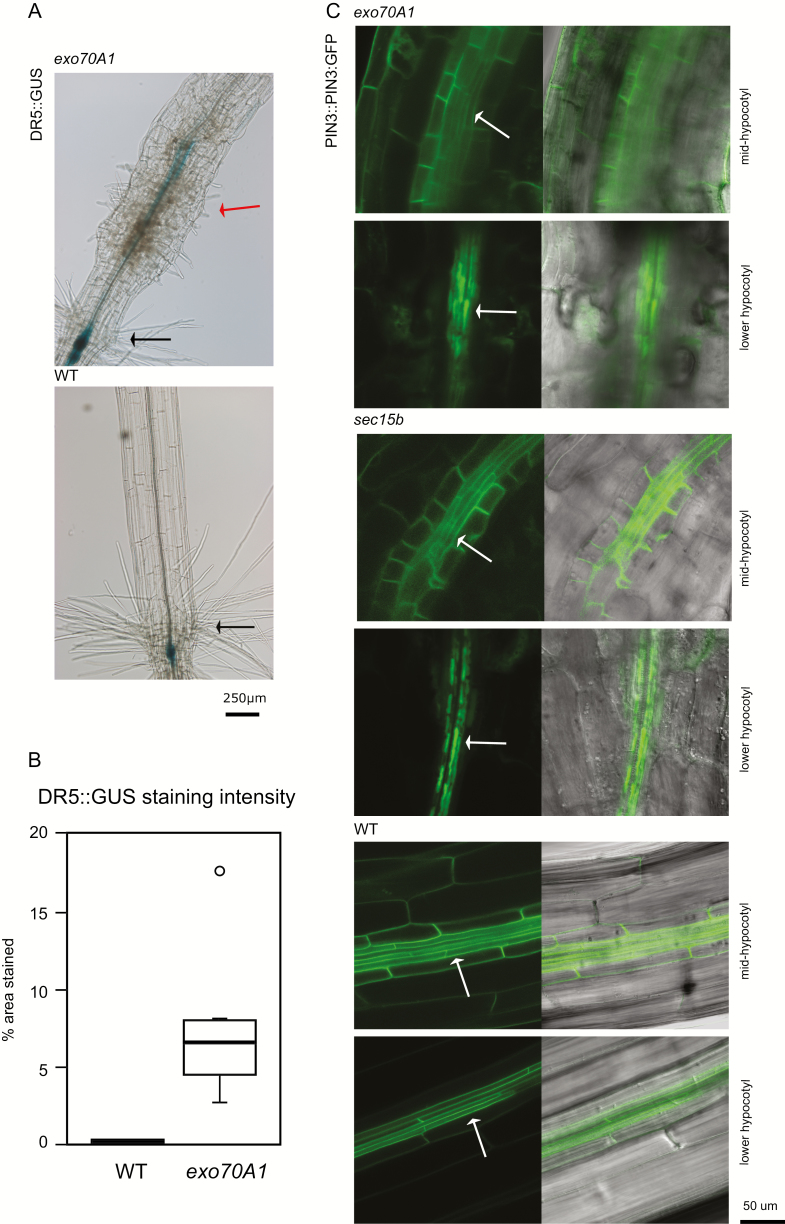
Auxin response reporter and auxin transporter localization in etiolated hypocotyls of exocyst mutants. (A) Activity of the DR5::GUS auxin response reporter in a 5-day-old etiolated TC *exo70A1* seedling and a WT control. Note the accumulation of the GUS signal in the stele of the ectopic collet-like region. Black arrows, collet hairs; red arrows, ectopic collet hair-like structures. (B) Quantitative comparison of GUS signal intensity in etiolated hypocotyls of WT and *exo70A1* seedlings. The difference between the two genotypes is highly significant for DR5::GUS (Wilcoxon test *P*<0.005, *n*=6). (C) Localization of PIN3–GFP in the middle and lower part of the hypocotyl in TC *exo70A1*, *sec15b*, and WT seedlings. The lower row corresponds to the ectopic collet-like region in mutants. White arrow, stele.

Since the EXO70A1 exocyst subunit is involved in auxin transport and in PIN1 and PIN2 recycling (Drdová *et al.*, 2013), we examined auxin distribution in mutant seedlings with ectopic collet-like structure using the auxin-responsive reporter DR5::GUS. While no signal was seen in WT hypocotyls, the stele of the ectopic collet-like region in *exo70A1* mutants showed pronounced GUS activity (Fig. 3A, B).

Next, we compared localization of GFP-tagged auxin transporter PIN3 (PIN3–GFP) in WT, *exo70A1*, and *sec15b* etiolated hypocotyls. While PIN3–GFP was membrane-localized along the entire stele in WT plants and in the anatomically normal part of mutant hypocotyls above the ectopic collet-like structure, the lower part of mutant hypocotyls including the ectopic collet-like structure accumulated PIN3–GFP signal preferentially in the vacuoles (Fig. 3C).

**Fig. 4.**
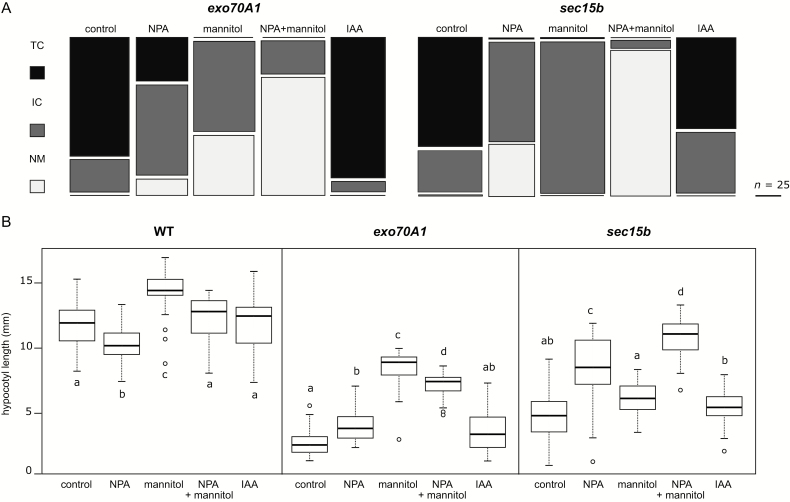
Effect of growth medium on phenotypic defects in *exo70A1* and *sec15b* mutant plants. (A) Mosaic diagrams displaying incidence of hypocotyl phenotypic classes among 5-day-old *exo70A1* and *sec15b* seedlings grown on different media types (NPA- and auxin-supplemented media with sucrose or mannitol). Column width reflects the number of plants counted; over 300 seedlings were evaluated in each experiment. NM, not malformed. Data from one experiment are shown; similar results were obtained in two independent replicates. Altogether, we found significant effects of medium type on the proportion of phenotypic classes in both mutants (pairwise χ^2^ test with Benjamini–Hochberg correction for multiple testing *P*<<0.0001; for IAA on *exo70A1* mutant *P*<0.05), except for the effect of IAA in *sec15b*, which did not significantly differ from the control. No hypocotyl malformations were observed in WT. (B) Boxplots summarizing the hypocotyl length in WT, *exo70A1*, and *sec15b* 5 d seedlings depending on cultivation media (over 70 plants per data point evaluated). Within each panel, the letters a, b, c, or d denote groups that did (different letters) or did not (same letters) differ significantly from each other as determined by series of *post hoc* pairwise Tukey tests with correction for multiple testing (at the level *P*<0.01—see Methods for statistical methodology). We also noticed significant interaction between genotype and cultivation medium type suggesting that medium composition had different effects on hypocotyl lengths in different genotypes.

To further examine possible auxin involvement in mutant phenotype development, we grew the seedlings on media containing the auxin transport inhibitor *N*-1-naphthylphthalamic acid (NPA; 5 µM) or auxin itself (20 nM indole-3-acetic acid (IAA)). NPA treatment partially alleviated the phenotypic effects of *exo70A1* and *sec15b* mutations (Fig. 4A). The NPA-grown mutant seedlings also had significantly elongated hypocotyl compared with non-treated controls (Fig. 4B). The NPA treatment had an opposite effect on WT seedlings, leading to slight reduction in hypocotyl length as reported previously (Jensen *et al.*, 1998). Treatment with IAA had little if any effect on either hypocotyl length or morphology in *exo70A1* or *sec15b* mutants (Fig. 4A, B).

**Fig. 5.**
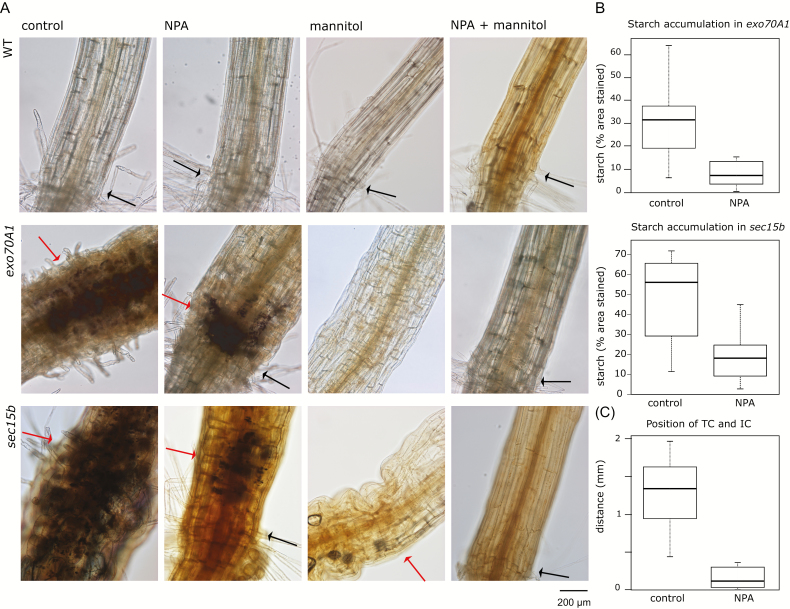
Influence of carbon source and NPA treatment on the accumulation of starch in etiolated hypocotyls of *exo70A1* and *sec15b*. (A) Hypocotyls of representative 5-day-old etiolated *exo70A1* and *sec15b* seedlings grown on media with sucrose or mannitol and with or without 5 µM NPA addition, stained for starch. No starch accumulation was observed in WT hypocotyls under these conditions. Black arrows, collet hairs; red arrows, ectopic collet hair-like structures. (B) Quantitative comparison of starch signal intensity in etiolated hypocotyls of *exo70A1* and *sec15b* seedlings grown on sucrose-containing media with and without NPA. Starch content was below detection limit in mannitol-grown plants. The effect of NPA in sucrose-grown plants is significant in both mutants (*sec15b*: Wilcoxon test *P*<0.003, *n*=10; *exo70A1:* Wilcoxon test *P*<0.03, *n*=8). (C) Effect of NPA treatment on the distance of ectopic collet-like structure from the true collet in the hypocotyl of *exo70A1*. The difference is statistically highly significant (Wilcoxon test *P*<0.0002, *n*=8 for each treatment).

Since altered starch distribution in ectopic collet-like region of *exo70A1* suggested possible changes in sugar metabolism, we also characterized the effects of varying carbon source in the medium on hypocotyl development. We grew seedlings on media with reduced metabolic carbon supply replacing sucrose with mannitol (mannitol medium). Mannitol medium caused slight elongation of WT hypocotyls as reported previously (Poupart *et al.*, 2005) and suppressed the effect of *exo70A1* and *sec15b* mutations with respect to both hypocotyl morphology and hypocotyl length (Fig. 4A, B). The suppression of mutant developmental defects was further enhanced by simultaneous addition of NPA to the mannitol medium (Fig. 4A), which also led to further prolongation of hypocotyl in *sec15b* mutants (Fig. 4B). The effects of NPA and mannitol medium thus appear to be additive, suggesting that an interplay of sugar- and auxin-based signaling, and possibly also sugar metabolism, may contribute to the mutant phenotype. Consistent with this hypothesis, *exo70A1* and *sec15b* seedlings grown on mannitol media with or without NPA did not accumulate any detectable starch in the hypocotyl, while mutant seedlings grown on NPA-supplemented sucrose media accumulated less starch in the ectopic collet-like region than those grown on sucrose media without NPA (Fig. 5A, B). The ectopic collet-like structure also developed significantly closer to the regular root–hypocotyl junction in NPA-treated plants than in non-treated ones (Fig. 5C).

### Cell identity in ectopic collet-like structure in *exo70A1* and *sec15b* mutants

**Fig. 6.**
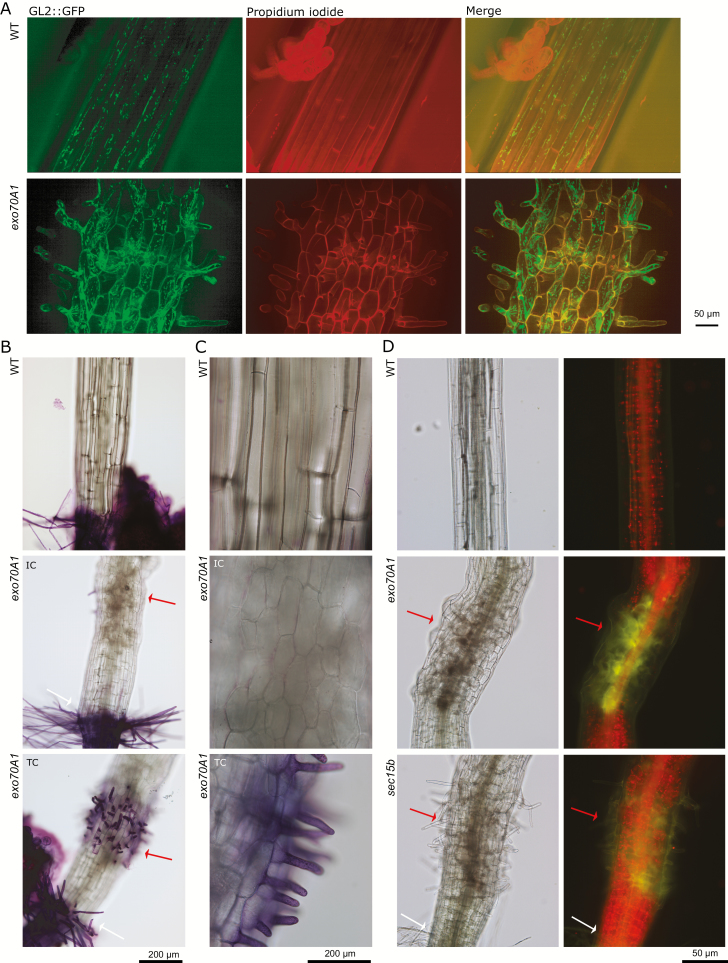
Disturbed cell identity in ectopic collet-like structures. (A) 3D-projection of *z*-stack confocal images showing expression of GL2::GFP in the cell files of WT and *exo70A1* etiolated hypocotyls (ectopic collet-like structure of a TC plant is shown for *exo70A1*). Cell walls were stained by propidium iodide. (B, C) Details of 5-day-old etiolated WT and *exo70A1* seedlings stained by toluidine blue, documenting cuticle permeability; two magnifications. (D) Reducer red autofluorescence of chlorophyll (observed using the GFP filter) in the ectopic collet-like region of *exo70A1* and *sec15b* seedlings grown 5 d in dark and 7 d in long day conditions. White arrows, collet; red arrows, ectopic collet-like structure.

The transcription factor GL2 (GLABRA2) is preferentially expressed in distinct cell files of hypocotyl epidermis or rhizodermis where it prevents the differentiation of stomata and root hair cells, respectively. GL2 expression is also absent in WT collet hairs (reviewed in Qing and Aoyama, 2012). Lack of *GL2* promoter activity can thus serve as a negative marker for collet hair identity. To investigate cell identity in the ectopic collet-like region, we introduced the GL2::GFP marker into *exo70A1* plants. Surprisingly, in the ectopic collet-like region of *exo70A1*, GL2::GFP was expressed in cell files like in the WT hypocotyl. Ectopic hairs were formed by both cells with and cells without the GFP signal (Fig. 6A), suggesting that GL2::GFP absence is not correlated with the presence of ectopic collet-like structures and formation of ectopic hairs.

Another proxy for cell identity is the surface characteristics of epidermal cells, since hypocotyl epidermis is covered by a cuticle, while rhizodermis is not. We used toluidine blue, which stains cells only in the absence of the cuticle (Tanaka *et al.*, 2004), to examine the surface of epidermal cells in the ectopic collet-like region. During short term staining of *exo70A1* mutants, the dye did not permeate enlarged cells of the IC phenotypic class. However, protruding ectopic hairs of TC seedlings were strongly labeled, although bodies of these cells remained unlabeled. Epidermal cells between regular root–hypocotyl junction and the ectopic collet-like region were covered with the cuticle as expected for hypocotyl, and were thus not permeated by toluidine blue (Fig. 6B, C).

Finally, we examined plastid development in the ectopic collet-like region since hypocotyl, unlike the collet, is a photosynthetic tissue characterized by abundant chloroplasts in light-grown plants. We transferred 5-day-old dark-grown *exo70A1* and *sec15b* seedlings to long day conditions and followed the distribution of chloroplasts based on the red chlorophyll autofluorescence after 7 d of de-etiolation. In the WT seedlings, the root–hypocotyl junction represents a border between chloroplast-rich hypocotyl and chloroplast-poor root (Scheres *et al.*, 1994). Mutant hypocotyls above and below the ectopic collet-like structure also developed abundant chloroplasts, but the cells in the ectopic collet-like region produced distinctly less chlorophyll (Fig. 6D).

We thus conclude that the ectopic collet-like region exhibits mixed cell identity with some characteristics of true collet.

## Discussion

The hypocotyl is a developmentally plastic structure that can re-program its ontogeny. Depending on environmental conditions, such as the light and contact with soil, it may function as the stem or can develop adventitious roots, thereby taking over the function of the root (Verstraeten *et al.*, 2014).

We describe a novel phenotypic deviation in Arabidopsis exocyst loss-of-function mutants characterized by the presence of an ectopic hair-bearing collet-like structure in the rootward part of etiolated hypocotyls. While such a phenotype was, to our knowledge, never described in WT plants or any other mutant, ectopic hair-like structures were reported in mutants overexpressing the *MIF1* gene (Hu and Ma, 2006) and those with modified GL2 function (Ohashi *et al.*, 2003). However, hairs covered large parts of mutants’ bodies in these cases. In contrast, ectopic hair-like structures of exocyst mutants localized to the narrow region close above the regular collet. The scarcity of data on development of the root–hypocotyl junction prevents clear-cut comparison with our observations; nevertheless, the aberrant region was morphologically similar to the true collet, and since the ectopic hairs were often formed on cells adjacent to each other, we propose to call this phenotypic deviation with developed ectopic hairs ‘twin-collet’ (TC) phenotype.

Because the exocyst EXO70A1 subunit is involved in the rootward polar auxin transport regulation and PIN1 and PIN2 recycling (Drdová *et al.*, 2013), we propose that perturbed auxin transport may contribute to these developmental aberrations. Consistent with this hypothesis, we observed increased auxin response in the ectopic collet-like region of *exo70A1* mutant and also mislocalization of PIN3–GFP to vacuoles in *sec15b* and *exo70A1* mutants. Furthermore, we found that treatment with NPA, an inhibitor of polar auxin transport, partially compensates the effect of *exo70A1* and *sec15b* mutants with respect to formation of ectopic collet-like structures, starch accumulation and hypocotyl elongation. We assume that NPA treatment inhibits auxin transport from the apex (Lehman *et al.*, 1996) and hence restores the balance in the auxin distribution in the hypocotyls of *exo70A1* and *sec15b* mutants resulting in the normalization of mutant phenotypic defects. This altogether highlights the role of auxin in the formation of the phenotypic defect.

Nevertheless, it is unlikely that the formation of the ectopic collet-like structure is induced solely by elevated auxin accumulation or activity in etiolated hypocotyls, since increase of auxin levels in Arabidopsis seedlings *per se* did not induce ectopic collets but caused development of adventitious roots along the hypocotyl (Boerjan *et al.*, 1995; King *et al.*, 1995). This suggests that auxin level modulation is just one part of the complex stimulus for the induction of the ectopic collet-like region. As another factor, the carbon metabolism or sucrose signaling also appear linked with the phenotypic deviations because the omission of sucrose from culture media (while keeping its osmolarity by mannitol) suppressed the formation of ectopic collet-like structures and starch accumulation, consistent also with sucrose being a starch precursor. Indeed, sucrose is an important morphogen in etiolated seedlings (Rolland *et al.*, 2006), known to affect auxin-related signaling, promote auxin accumulation (Stokes *et al.*, 2013), induce expression of PIN7 and stimulate rootward auxin transport from the cotyledons (Lilley *et al.*, 2012). Since the omission of sucrose and NPA treatment acted in an additive manner in *exo70A1* and *sec15b*, we hypothesize that effects of sucrose are at least partly independent from those of auxin transport and signaling.

Detailed analysis of TC seedlings revealed that the area between the ectopic collet region and true collet possesses all the characteristics of the normal hypocotyl, such as two complete cortical cell layers, cuticle on the surface of epidermal cells and the ability to produce a high amount of chlorophyll after light treatment. The ectopic collet-like region of TC plants, on the other hand, displays mixed cell identity and is morphologically similar to the true collet. Also, the ectopic hairs of TC plants generally did not possess continuous cuticle, albeit plants exhibiting the less severe IC phenotype retained cuticle continuity. The defect extends into the organization of inner tissues (cortex, endodermis), where incomplete cell layers in the cortex were observed, reminding us of the typical organization of cell layers in the true collet region (Lin and Schiefelbein, 2001). Similar to the true collet, the defective area also exhibits reduced ability to produce chlorophyll in seedlings moved from the dark to the light.

There are currently no positive developmental markers for the Arabidopsis collet region. We propose that the GL2 transcription factor may be considered as a negative marker for the hair-bearing cells of the true collet. GL2 is specifically expressed in atrichoblasts where it represses root hair and stomata formation, and it is not expressed at the root–hypocotyl junction, allowing collet hair formation from all epidermal cells (see Qing and Aoyama, 2012). Surprisingly, *exo70A1* mutants expressed GL2::GFP continuously in the cell files through the entire hypocotyl, including hair-bearing cells of the ectopic collet-like region. Thus, hairs of the ectopic collet-like structure appear to be initiated from epidermal cells with original hypocotyl identity; the change in cell fate obviously occurs downstream from the step controlled by GL2.

We propose that the ectopic collet-like region develops post-embryonically, since observed phenotypic deviations occur only in dark-grown seedlings and the penetrance of the defect can be affected by cultivation temperature. The presence of many collapsing cells in the ectopic collet-like region further suggests that incomplete cell layers form post-embryonically rather than during primary embryonic development. Also the altered pattern of adventitious roots produced from the hypocotyls points toward post-germination effects of the studied mutations.

The root–hypocotyl junction is an important transition zone in angiosperms and its correct organization is crucial for plant fitness. Here we document a new role of the exocyst complex, intimately interlinked with sugar metabolism and auxin transport and signaling, in these processes. After years of neglect, the fascinating region of root–hypocotyl junction clearly deserves future experimental attention.

## Supplementary data

Supplementary data are available at *JXB* online.

Fig. S1. Graphical visualization of s*ec15b-1* and *sec15b-2* insertions in *SEC15B* gene and promoter region, and RT-PCR confirmation of the absence of *SEC15B* mRNA in *sec15b-1* and *sec15b-2* mutant lines.

Fig. S2. Phenotypic defects of etiolated hypocotyls in exocyst mutants.

Table S1. Primers used for genotyping and mRNA level analysis of *sec15b-1* and *sec15b-2* mutant lines.

Table S2. Formation of adventitious roots in etiolated seedlings upon transfer to light.

Supplementary Figures S1-S2 and Tables S1-S2

## Abbreviations:

exo70A1: *exo70A1-2*
IAA: indole-3-acetic acid
IC: irregular cells
GFP: green fluorescent protein
GL2: glabra 2
GUS: β-glucuronidase
NPA: *N*-1-naphthylphthalamic acid
PIN: auxin efflux carrier
ROI: region of interest
sec15b: sec15b-1
TC: twin collet
WT: wild type
