# Supplementary material for: Developmental plasticity of Arabidopsis hypocotyl is dependent on exocyst complex function
Source: J Exp Bot. 2019 Jan 14;70(4):1255–65. doi: 10.1093/jxb/erz005 (PMC6382343; doi:10.1093/jxb/erz005)
Supplement: Supplementary Figures S1-S2 and Tables S1-S2 [file erz005_suppl_supplementary_figure_s1-s2_table_s1-s2.pdf]

Table S1. Primers used for genotyping and mRNA level analysis of *sec15b-1* and *sec15b-2* mutant lines.

Primer	Sequence (5' – 3')	Purpose
SEC15BPROM-RP	GTTGAATTCCTATGGTTTCTAGAAAAC	genotyping of SALK_130663 line
SEC15BPROM-LP	AAGCATCCGTTTCAGCGTTGAC	genotyping of SALK_130663 line
LBNEW	GAACAACACTCAACCCTATCTCGGGC	genotyping of SALK_130663 line
SEC15BRIK-RPBF	TTGGATCCATATGCAATCGTCGAAAGGA	genotyping of RATM15-1183-1_H line
SEC15BRIK-LPBIR	CAGTAAGAGATGATTAGCCGTC	genotyping of RATM15-1183-1_H line
DS52A	TCCGTTCCGTTTTCGTTTTTTAC	genotyping of RATM15-1183-1_H line
NPT_LP	TCCGAGTACGTGCTCGCTCGATGC	primers specific to kanamycin resistance gene
NPT_RP	GCTTGGGTGGAGAGGCTATTCGGC	primers specific to kanamycin resistance gene
SEC15BRIK-RPBF	TTGGATCCATATGCAATCGTCGAAAGGA	mRNA level analysis
SEC15BRIK-LPBIR	CAGTAAGAGATGATTAGCCGTC	mRNA level analysis
ACT7-FWD	GCCGATGGTGAGGATATTCAGC	mRNA level analysis
ACT7-REV	GAAACTCACCACCACGAACCAG	mRNA level analysis

Table S2. Formation of adventitious roots in etiolated seedlings upon transfer to light

Genotype	Plants No	Roots from hypocotyl	Roots from collet	Roots from collet (%)
WT *SEC15b*	42	51	11	18
sec15b	56	6	40	87
WT *EXO70A1*	21	14	1	7
exo70A1	39	1	28	97

5-day-old etiolated seedlings were transferred to long day conditions and the presence of emerged adventitious roots was scored after 7 days. Sister WT segregants from the last backcross were evaluated for each genotype. For each gene, the mutation significantly affects adventive root location (χ^2 test with Benjamini-Hochberg correction for multiple testing $p<<0.001$). The effect on overall adventitious root number is marginally significant for *sec15b* (χ^2 test with Benjamini-Hochberg correction $p = 0.049$) and non-significant for *exo70A1*. GLMM analysis further confirmed that the distribution of adventitious roots is significantly affected by the genotype ($p = 0.027$ for WT vs. *sec15b*, $p = 0.007$ for WT vs. *exo70A1*), as well as by the location (collet vs. hypocotyl; $p<0.001$ for either genotype), and indicated significant interaction between genotype and location ($p<0.001$ for either genotype).

Figure S1.
(A) Graphical visualization of *sec15b-1* and *sec15b-2* insertions in *SEC15B* gene and promoter region.
(B) RT-PCR confirmation of the absence of *SEC15B* mRNA in *sec15b-1* and *sec15b-2* mutant lines.

(A)

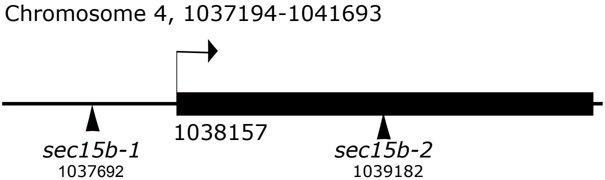

(B)

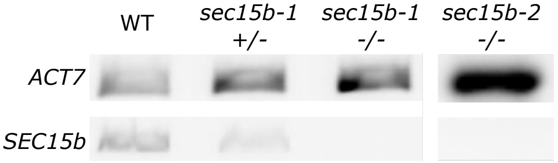

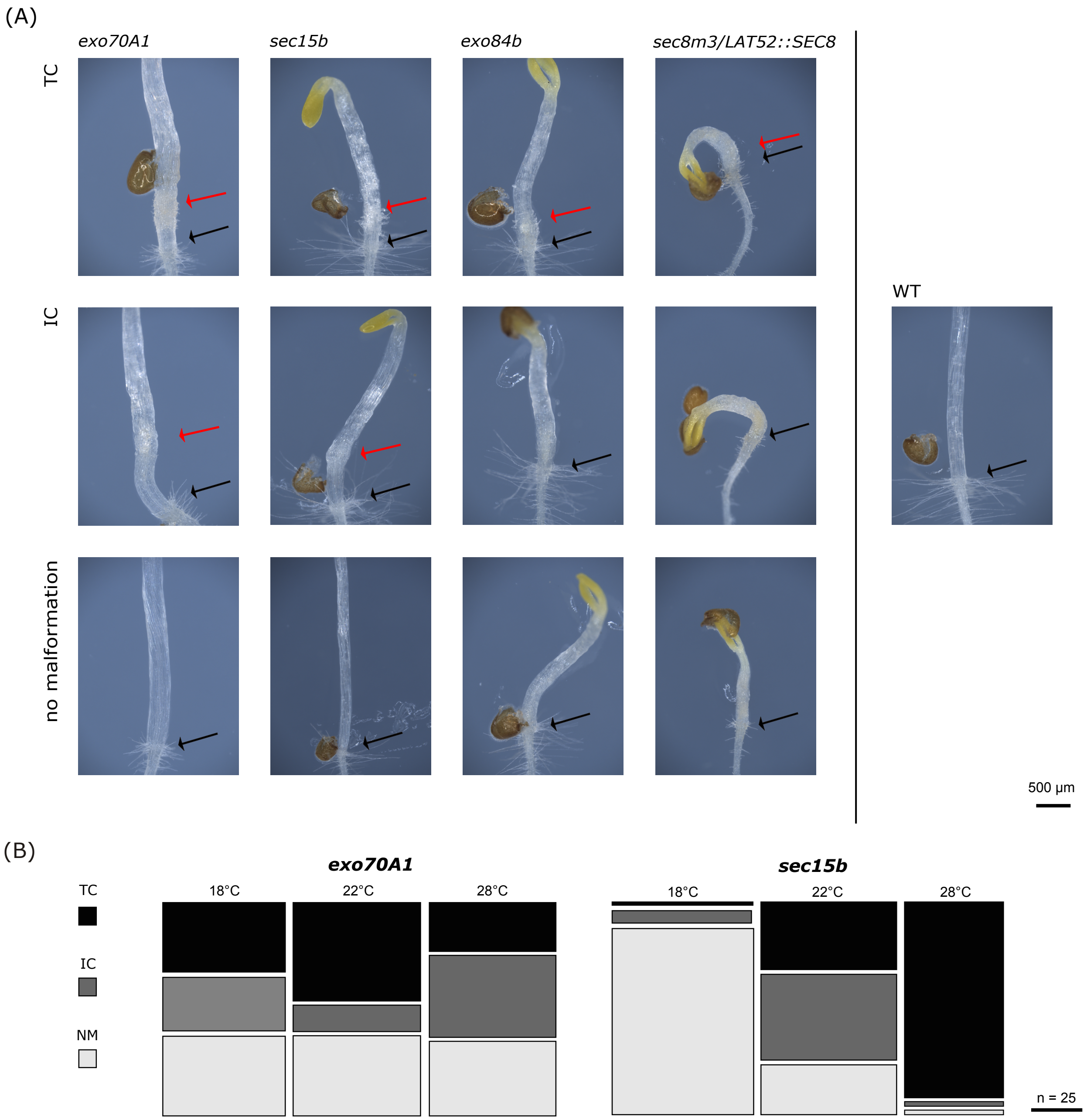

(A)
exo70A1
sec15b
exo84b
sec8m3/LAT52::SEC8
TC
IC
no malformation
WT
500 µm
(B)
exo70A1
sec15b
TC
IC
NM
18°C
22°C
28°C
n = 25

Fig. S2. Phenotypic defects of etiolated hypocotyls in exocyst mutants.

(A) Typical phenotypic deviations - ectopic collet-like structures with or without developed hairs (phenotypes TC and IC) and shortened hypocotyls without malformations in 5 days old *exo70A1*, *sec15b*, *exo84b* and *sec8m3/LAT52::SEC8* seedlings. Black arrows - collet hairs, red arrows - ectopic collet-like structures. A WT seedling is shown alongside for comparison. (B) Effect of cultivation temperature on the proportion of the phenotypic deviations in *exo70A1* and *sec15b* seedlings. Mosaic diagrams display fraction of individuals in the phenotypic categories among seedlings developed at the indicated temperature (NM – no malformation). In total 233 *exo70A1* and 452 *sec15b* mutant plants were evaluated; temperature effects were reproducibly highly significant only for the *sec15b* mutant (pairwise χ^2 test with Benjamini-Hochberg correction for multiple testing $p<<0.001$ for each temperature combination). Column width reflects the number of seedlings counted. Data from one experiment are shown; similar results were obtained in 3 experiments. All seedlings were 5 days old. No hypocotyl defects were ever observed in WT seedlings under these culture conditions.
